# Possible Complications of Ureteroscopy in Modern Endourological Era: Two-Point or “Scabbard” Avulsion

**DOI:** 10.1155/2014/308093

**Published:** 2014-12-28

**Authors:** Andrius Gaizauskas, Marius Markevicius, Sergejus Gaizauskas, Arunas Zelvys

**Affiliations:** ^1^Vilnius University, Siltnamiu 29, LT-01430 Vilnius, Lithuania; ^2^Vilnius University, Universiteto 3, LT-01122 Vilnius, Lithuania; ^3^Republic Vilnius University Hospital, Siltnamiu 29, LT-01430 Vilnius, Lithuania; ^4^Vilnius University, Santariskiu 2, LT-08861 Vilnius, Lithuania

## Abstract

Indication has led ureteroscopy to be a worldwide technique, with the expected appearance of multiple types of complications. Severe complications are possible including ureteral perforation or avulsion. Ureteral avulsion has been described as an upper urinary tract injury related to the action of blunt trauma, especially from traffic accidents, being the mechanism of injury, the result of an acute deceleration/acceleration movement. With the advent of endourology, that term is also applied to the extensive degloving injury resulting from a mechanism of stretching of the ureter that eventually breaks at the most weakened site, or ureteral avulsion is referred to as a discontinuation of the full thickness of the ureter. The paper presents a case report and literature review of the two-point or “scabbard” avulsion. The loss of long segment of the upper ureter, when end-to-end anastomosis is not technically feasible, presents a challenge to the urological surgeon. In the era of small calibre ureteroscopes these complications, due to growing incidence of renal stones will become more and more actual. Our message to other urologists is to know such a complication, to know the ways of treatment, and to analyse ureteroscopic signs, when to stop or pay attention.

## 1. Introduction

Ureteroscopy is a diagnostic and treatment modality, used for different ureteral and renal pathologies. Ureteroscopy was first carried out in 1929 by Young and Mckay using a cystoscope in grossly dilated ureter [1, 2], but it was not until the late 1970s that the rigid ureteroscope was used [3].

Since its clinical introduction in 1982 by Perez-Castro Ellendt and Martinez-Pineiro, ureteroscopy has experienced an impressive development due to the technical improvements of new and smaller urological armamentarium [3, 4]. In the last decade ureteroscopy has become an outstanding breakthrough in the diagnosis and treatment of different ureteral and renal problems.

Today it is increasingly used in the management of the common ureteral stones, and such frequent indication has led ureteroscopy to be a worldwide technique, with the expected appearance of multiple types of complications, some of which are severe, including ureteral perforation or avulsion, bleeding, and urinary tract infection [4].

The term ureteral avulsion has been described as an upper urinary tract injury related to the action of blunt trauma, especially from traffic accidents, being the mechanism of injury, the result of an acute deceleration/acceleration movement [4, 5]. With the advent of endourology, that term is also applied to the extensive degloving injury resulting from a mechanism of stretching of the ureter that eventually breaks at the most weakened site [4], or ureteral avulsion is referred to as a discontinuation of the full thickness of the ureter [6]. The first cases were reported by Hart et al. [4, 7] in 1967 and Hodge et al. [4, 8] in 1973, both after difficult manipulation of a ureteral stone with Dormia basket.

Ureteroscopy has gradually become a major technique for the diagnosis and treatment of lesions of both the ureter and the intrarenal collecting systems [9].

**Figure 1:**
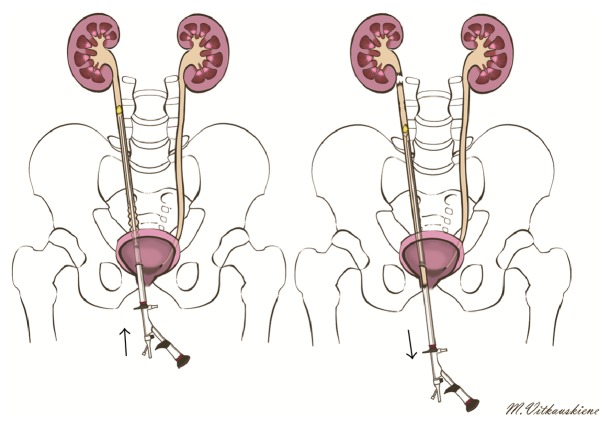
Scheme of complete ureter avulsion.

**Figure 2:**
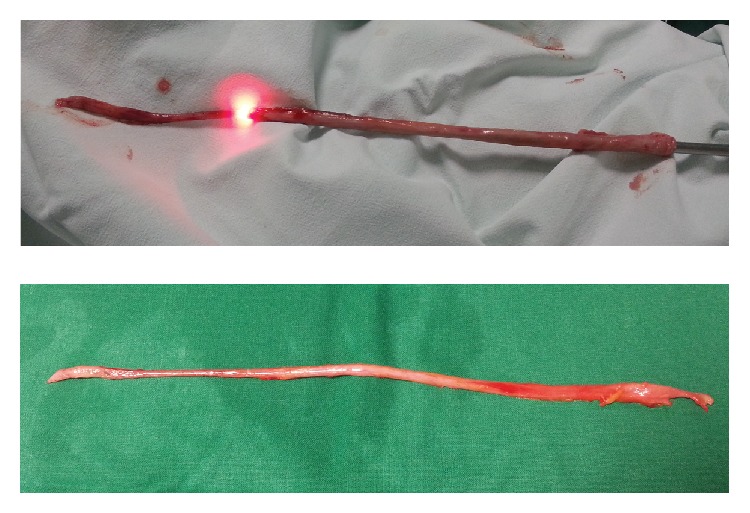
Two-point or “scabbard” avulsion.

*Case of “Scabbard” Avulsion*. A 52-year-old male came to the emergency department with renal colic. In emergency department performed kidney ultrasound revealed right hydronephrosis. Then a plain abdominal radiography was performed that revealed X-ray positive, 5 mm stone in the upper third of the ureter. Patient was sent home and prescribed tamsulosin 0.4 mg and analgesics. After two weeks patient came back to hospital, for urological consultation. The same stone was found in the same position, and ultrasound showed hydronephrosis in the right kidney. Patient was hospitalized in urological department, for ureteroscopy and stone extraction. A semirigid ureteroscope 9.8 Fr was used. Ureteroscopy started with insertion of ureteroscope to urethra and bladder. A guidewire was inserted to the ureter up to the right kidney and then scope inserted to right ureter alongside guidewire. A little bit of tight sensation was felt inside intramural ureter. Some pressure was needed to move the scope up to the stone. No ureteral stricture was seen, and ureter was not tight or narrow. Stone was found in the upper third of the ureter. Stone was caught by basket and slowly moved down the ureter. Near the entrance of bladder an X-ray contrast material was injected, to be sure that there is no contrast leak out of ureter. Going out of ureter slowly, image disappeared and scope easily moved out of urethra. From the distal part of ureteroscope up to middle part, we found the whole ureter firmly engaged to the ureteroscope as a “scabbard” avulsion of ureter was diagnosed (Figures 1 and 2).

**Figure 3:**
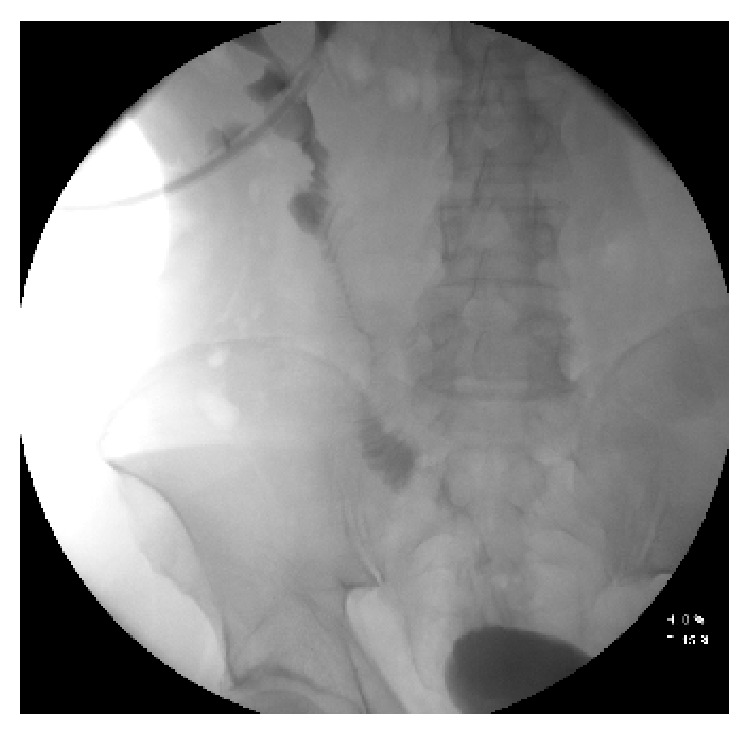
Nephrostogram.

At the same day lumbotomy was performed and proximal part of ureter just below pyeloureteric junction was tied. Ureter was sent for histological evaluation. Pathologist has found chronic ureteritis and fibrotic tissue (like scar) in the distal part of ureter, without elastic fibre. After operation patient remembered that he had a trauma of right iliac side after which was a period of haematuria. That can explain histological findings. After four months a laparotomy was performed, and ileum interposition was performed, with anastomosis end-to-end with renal pelvis and urinary bladder. 30 cm of ileum was used. Postoperatively no complications occurred. On the 16th postoperative day patient was discharged. During hospitalization, nephrostogram was performed, that revealed no obstruction or contrast leakage (Figure 3). Ultrasound revealed no hydronephrosis.

**Figure 4:**
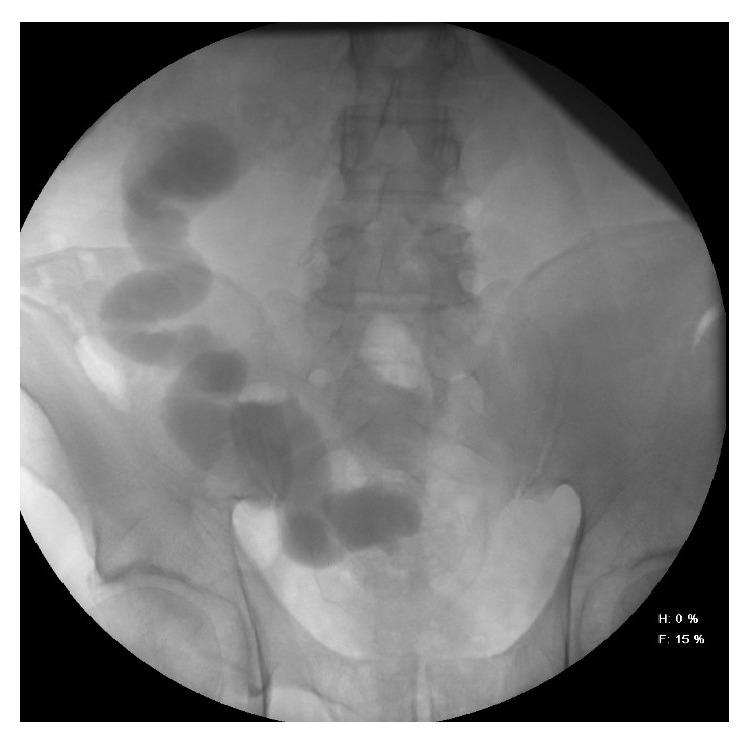
Voiding cystoureterogram.

At home he was prescribed to use nitrofurantoin microcrystals 100 mg once a day before sleep. During follow-up of more than 6 months, routine diagnostic investigations revealed no hydronephrosis, and intravenous urography showed identical function of both kidneys. During voiding cystoureterogram, I° degree of vesicoileal reflux was found (contrast went up the ileum but did not reach the kidney) (Figure 4). From anamnesis of the patient, he had no signs of urinary system infection. Sometimes he urinates ileum mucus.

## 2. Discussion

Complete avulsion of the ureter is a catastrophic complication and is fortunately exceptionally rare [11]. Avulsions are undoubtedly the most serious complication that may occur during a URS. Ureteral avulsion during ureteroscopic stone management has been reported in 0 to 3.75% of series and fortunately does not seem to be increasing, despite an increase in the number of ureteroscopic cases being performed worldwide [6, 9, 12–16].

Complications of ureteroscopy have been categorized into minor and major events: minor complications include asymptomatic ureteral perforations, ileus, and fever, whereas major complications, which are more often associated with stone extractions, include tears, perforations during basketing, and, rarely, avulsions, intussusception, and sepsis. Necrosis of ureteral segments after ureteroscopy for stone removal has also been reported [17–20]. Complications are considered major if operative intervention is required or if they are life-threatening. Major complications of ureteroscopy may have severe and lasting consequences [9]. Minor complications are those that are adequately managed nonoperatively. Generally open or laparoscopic operative intervention is almost always required in major types of complication and the basic aim is to restore the ureteral continuity [4, 20]. Intraoperative complications were especially associated with proximal-ureteral calculi. Geavlete and colleagues reported the intraoperative complication rate was 5.6% for proximal calculi and 3.6% for distal stones [9]. For proximal stones there is possibility to use extracorporeal shock wave lithotripsy (SWL), and fortunately the frequency of reported complications following SWL of stones in the ureter is low but in the range of 0–6% [21].

While reviewing literature we did not find specific classification of ureteral avulsion, we considered idea to propose and describe types of avulsion and to classify them into one-point and two-point ureteral avulsion (Table 1), because different situations require different treatment tactics, and while reading articles it is difficult to understand what kind of avulsion is mentioned. Also talking about percentage of avulsion that occurred we do not see a magnitude of the problem.

Lithotripsy method during ureteroscopy may have effect on complication rate. Georgescu with colleagues in large study demonstrated that while using electrohydraulic fragmentation they had 3.9% complication rate. In cases when pneumatic or laser lithotripsy was used they had 3% and 2.8% complication rate. But the difference among those methods was not statistically significant (*P* = 0.3282) [22]. Still we think that lithotripsy method can have effect on mechanical or thermal ureter injury which may cause ischemia, perforation, or bleeding but not avulsion. Some authors maintain that improvements in ureteroscope design (development of small calibre semirigid and flexible deflectable ureteroscopes and development of diminutive lithotripsy probes) and technique have determined the success of diagnostic and therapeutic ureteroscopy and reduced the incidence of major complications [9, 23, 24]. Equal to this statement a study by Taie and colleagues stated that, with increasing surgeon's experience and evolving devices, the rates of ureteral perforation and avulsion have decreased from 3.3% to 0.5% and from 1.3% to 0.1%, respectively [18, 25]. However despite significant technologic advances, with the wide application of percutaneous nephroscopes, ureteroscopes, and endoscopic stone extractors, the incidence of iatrogenic ureteral avulsion tends to grow year by year [6, 9, 24]. We are talking not about percentage of avulsion but about the cases that occur and about urologist that has to tend with that kind of complication.

The mechanism of complete avulsion of ureter in patients can occur during advancement of ureteroscope through a very tight ureteric stricture up to the level where the stone is tightly impacted. The tight grip of fibrous stricture on the body of scope can cause the first avulsion at distal third of ureter during its advancement. The second avulsion then occurs during withdrawal of ureteroscope, at the site where stone is impacted and this area was further weakened by the microtrauma created by the shockwave lithotripsy or stone impaction. Discrepancy between ureteroscope size and calibre of the patient's ureter is another factor of possible avulsion [11]. Ureteral injuries can occur throughout the entire length of ureter, and more severe injuries are associated with the proximal ureter, which has less muscular support and fewer mucosal cell layers than the distal or intramural ureter [26]. Among the potential factors involved in the pathogenesis of ureteral avulsion, the presence of an anomalous ureter, either due to a diseased area or to previous endourologic manipulations, is an important antecedent in the majority of cases [8]. Another thing that can cause complete avulsion of the ureter close to the bladder wall may be attributed to extensive oedema in the lower ureter fastening the endoscope to this part of the ureter [27]. Incarcerated stones also cause ureteral mucosa inflammation, oedema, ureteral tortuosity and stenosis, increased fragility, and reduced elasticity of ureteral tissue [6].

In recent publication of Ulvik and Wentzel-Larsen it was explained that the forces needed to advance and retract the ureteroscope were considerably larger in the upper ureter than in the lower. It is conceivable that the resistance between the ureteral wall and the ureteroscope will increase as the area of contact between them becomes larger during the advancement of the instrument. Interestingly, the forces needed for retraction of the ureteroscope were significantly smaller than the forces needed for insertion at all levels of the ureter, except at the level of the iliac vessels. The insertion of the ureteroscope dilates the ureter and may thereby facilitate the subsequent retraction [27]. But retrospectively analysing our complication, the forces moving the scope up the ureter to the calculi, when distal part of ureter avulsed, changed from large power needed to advance to low power. That happened because ureter tightly compressed the scope and avulsed. It would be interesting to understand and to know when we use more power than it should be used. While moving ureteroscope down the ureter, in normal circumstances as Ulvik and Wentzel-Larsen [27] described we need less force to retract scope than to advance. During our performed operation we needed much more force, during retraction of scope down. At that moment we needed to stop and think of the reason why it is so. But as we thought that we saw recessing of ureter surface, we did not take into consideration that distal part of ureter is already avulsed and fixed to the scope.

Conversely, in the cases with the ureteroscope already up the ureter, there is no limit to the amount of force that may be applied. The degree of force applied is limited only by the sense and experience of the operator [14].

This complication is more common in the proximal ureter where the muscular and mucous layers are less present. However, when a ureter becomes less resistant and elastic, due to previous traumas or endourological procedures, it may be subject to avulsions in any of its segments [12]. After operation patient remembered having a trauma of right iliac part of abdomen, and histology revealed fibrosis of the distal part of ureter that fixed to the scope during operation and did not let the scope move easily.

The loss of long segment of the upper ureter, when end-to-end anastomosis is not technically feasible, presents a challenge to the urological surgeon. Autotransplantation is often the preferred treatment [28]. Other possible techniques used for ureteral reconstruction include psoas hitch, Boari flap, or a combination of both, ileal interposition, transureteroureterostomy, permanent nephrostomy, appendix or colon interposition, renal descensus, ureterocalicostomy, and pyeloureterostomy plus greater omentum investment outside the avulsed ureter, and the last easiest way is to perform nephrectomy [4, 6, 14, 15, 23, 28, 29].

Since Hardy in 1963 described the use of renal autotransplantation in ureteral injury, this method became one of the treatment modalities in irreversible ureteral injuries [23]. Use of the appendix as a ureteral substitute began in 1912 by Melinkoff but it did not become a popular method at that time [11].

In modern era, when we are trying to find answers to all possible questions we have guidelines that can help us in resolving even that kind of complications. EAU guidelines indicate reconstruction options by site of injury (Table 2) [10]. This table is created not for avulsions but for ureteral trauma. Ureteral avulsion is complication of ureteroscopy, so it is iatrogenic trauma and this table is very helpful when urologist is searching for answers and possibilities. This is evidence based recommendation that offers the best possible opportunity.

Ileal interposition is a very rare operation. During literature review it was possible to find only articles which were written between 1960 and 1990. However we have found several newer articles that described their analysis of patients for whom ileal interposition was performed. Some of these patients had follow-up as long as 6.04 years and mean age was 48.1 years [30, 31]. They performed retrospective analysis and have found that ileal ureter substitution remains an effective treatment for patients with complex ureteral strictures or injuries [31]. Overall the complication rate is low. Most postoperative complications were minor in nature, including pyelonephritis, fever of unknown origin, neuroma, hernia, recurrent urolithiasis, and deep venous thrombosis. Major complications include anastomotic stricture, ileal graft obstruction, wound dehiscence, and chronic renal failure. Overall patients did not experience worsening renal function after the procedure in two retrospective analyses, but another showed that serum creatinine decreased or remained stable in 74.7% of cases. Some of the patients experienced hyperchloremic metabolic acidosis [30–32]. But a conclusion was given that ileal interposition is safe and effective operation for properly selected patients.

Prophylaxis of ureteral avulsion concurs especially with the endoscopic skill of the urologist and the adherence to some basic rules, such as using a small ureteroscope or avoiding Dormia basket retrieval of the stone in cases of large calculus or partial view of the area where the calculus is impacted [4]. It is important to address this discrepancy in the scope and ureteric lumen size by prior dilatation of the distal ureteral stricture after the passage of the guidewire. A balloon dilator under fluoroscopic guidance could easily dilate the stricture to 15–18 Fr, thereby avoiding the ripping effect of the fibrotic ring on the ureteroscope [2].

Other practical suggestions are (1) that prior to insertion of ureteroscope to the ureter insert a guidewire, because then you will not have full contact between scope and ureter lumen; (2) if the urologist finds it difficult to advance the ureteroscope despite full visualization of the lumen and senses that the scope feels tight in the ureter, the scope should be withdrawn immediately and either replaced with a smaller calibre scope and reinserted after ureteral dilation or ureteroscopy should be repeated on a later occasion after placement of a ureteral stent to allow ureteral dilation; (3) if you see ureteral stricture, dilatation prior to the insertion of scope or incision of stricture should be performed; (4) if you plan to perform proximal ureteroscopy lubricant should be applied along the entire length of the shaft; and (5) if you feel that scope is impacted or you need more power for extraction of scope, a second semirigid ureteroscope should be passed into the bladder beside the first and the situation is evaluated [9, 14].

## 3. Conclusion

Two-point or “scabbard” ureter avulsion is extremely rare ureteroscopy complication, which terrifies urologists performing ureteroscopic procedures. Today we use semirigid and flexible small calibre ureteroscopes. These complications, due to growing incidence of renal stones and cases or ureteroscopes, will become more and more actual. Our message to other urologists is to know such a complication, to know the ways of treatment, and to analyse ureteroscopic signs, when to stop or pay attention. The best way of resolving that kind of complication would be ileal interposition or laparoscopic nephrectomy with or without autotransplantation.

## Figures and Tables

**Table 1:** Classification of ureteral avulsion.

One-point ureteral avulsion	Two-point ureteral avulsion (discontinuity of ureter)
Ureteral avulsion, when ureter rupture is in the distal part of ureter	Avulsion of ureter from proximal to middle part of ureter (upper third)
Ureteral avulsion, when ureter rupture is in the middle part of ureter	Avulsion of middle part of ureter (middle third)
Ureteral avulsion, when ureter rupture is in the proximal part of ureter	Avulsion of distal part of ureter (lower third)
Avulsed only endothelium (there is no discontinuation of whole ureter walls)	Avulsion of proximal and middle part of ureter (upper and middle third)
	Avulsion of middle and distal part of ureter (middle and lower third)
	Avulsion of whole ureter (scabbard avulsion)

**Table 2:** Reconstruction options by site of injury [10].

Site of injury	Reconstruction options
Upper ureter	Ureteroureterostomy Transureteroureterostomy Ureterocalicostomy

Mid ureter	Ureteroureterostomy Transureteroureterostomy Ureteral reimplantation and a Boari flap

Lower ureter	Ureteral reimplantation Ureteral reimplantation with a psoas hitch

Complete	Ileal interposition graft Autotransplantation
